# Therapeutic Targets for DOX-Induced Cardiomyopathy: Role of Apoptosis vs. Ferroptosis

**DOI:** 10.3390/ijms23031414

**Published:** 2022-01-26

**Authors:** Hiroki Kitakata, Jin Endo, Hidehiko Ikura, Hidenori Moriyama, Kohsuke Shirakawa, Yoshinori Katsumata, Motoaki Sano

**Affiliations:** Department of Cardiology, Keio University School of Medicine, Tokyo 160-8582, Japan; kitkat@keio.jp (H.K.); jinendo@keio.jp (J.E.); ikurahidehiko@keio.jp (H.I.); h.moriyama@keio.jp (H.M.); shirakawa19840905@gmail.com (K.S.); goodcentury21@gmail.com (Y.K.)

**Keywords:** doxorubicin, cardiotoxicity, doxorubicin-induced cardiomyopathy, apoptosis, ferroptosis

## Abstract

Doxorubicin (DOX) is the most widely used anthracycline anticancer agent; however, its cardiotoxicity limits its clinical efficacy. Numerous studies have elucidated the mechanisms underlying DOX-induced cardiotoxicity, wherein apoptosis has been reported as the most common final step leading to cardiomyocyte death. However, in the past two years, the involvement of ferroptosis, a novel programmed cell death, has been proposed. The purpose of this review is to summarize the historical background that led to each form of cell death, focusing on DOX-induced cardiotoxicity and the molecular mechanisms that trigger each form of cell death. Furthermore, based on this understanding, possible therapeutic strategies to prevent DOX cardiotoxicity are outlined. DNA damage, oxidative stress, intracellular signaling, transcription factors, epigenetic regulators, autophagy, and metabolic inflammation are important factors in the molecular mechanisms of DOX-induced cardiomyocyte apoptosis. Conversely, the accumulation of lipid peroxides, iron ion accumulation, and decreased expression of glutathione and glutathione peroxidase 4 are important in ferroptosis. In both cascades, the mitochondria are an important site of DOX cardiotoxicity. The last part of this review focuses on the significance of the disruption of mitochondrial homeostasis in DOX cardiotoxicity.

## 1. Introduction

Drug therapy for cancer has made remarkable progress in recent years with the development of molecular-targeted drugs and immune checkpoint inhibitors alongside conventional chemotherapy. As a result, the survival rate of cancer patients has markedly improved [1]. As the prognosis of cancer patients improves, the issue of increased risk of developing cardiovascular disease due to anticancer drug therapy is rapidly gaining attention [2,3,4]. In fact, for breast cancer patients with a history of cardiovascular disease, mortality due to cardiovascular disease is higher than mortality due to breast cancer more than five years after the diagnosis of breast cancer [5]. Conversely, heart failure patients are at an increased risk of cancer [6]. In addition, as the population ages and cancer treatment advances, the number of patients with both conditions has been increasing [7]. In this context, the field of oncocardiology has become increasingly important in recent years [8].

Heart failure associated with anticancer drugs is classified into two categories: whether the mechanism of cardiotoxicity is irreversible (type I) or reversible (type II). As Table 1 shows, irreversible myocardial injury caused by anthracyclines such as doxorubicin (DOX) is defined as type I. In contrast, reversible myocardial injury caused by trastuzumab, a human epidermal growth factor receptor 2 (Her2) inhibitor, and later bevacizumab, sunitinib, and sorafenib, is defined as type II [2,9]. Generally, type II drugs cause cardiomyocyte dysfunction but not myocardial necrosis, and their cardiotoxicity is considered reversible [9]. This review will introduce the molecular mechanisms of DOX cardiotoxicity and anthracyclines that cause type I myocardial injury and will discuss possible countermeasures.

Anthracyclines are a class of drugs used in cancer chemotherapy and are derived from *Streptomyces* *peucetius var. caesius* [10]. Anthracyclines include DOX, daunorubicin, epirubicin, idarubicin, pirarubicin, and amrubicin. They were developed in the 1970s and are widely used in clinical practice. They inhibit DNA topoisomerase II (TopII) and intercalate between base pairs of DNA strands, inhibiting DNA synthesis, replication, and transcription and suppressing cell proliferation. Among them, DOX (sold under the brand name Adriamycin) is one of the most widely used anticancer drugs in cancer treatment [11]. It has been used for hematopoietic tumors and solid tumors, such as breast cancer, resulting in improved cure rates and quality of life for cancer patients [12]. However, despite its outstanding efficacy, the risk of DOX cardiomyopathy, which develops in a cumulative dose-dependent manner, is often experienced as a limitation to continued treatment [13].

**Figure 1:**
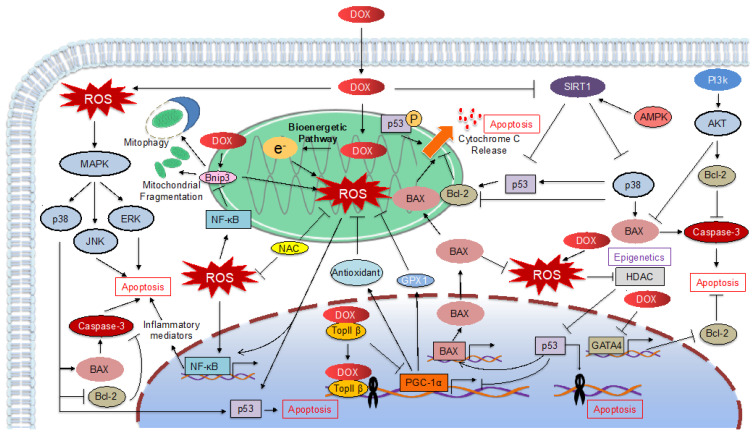
Schematic representation of Doxorubicin (DOX)-induced apoptosis pathway. DOX, doxorubicin; ROS, reactive oxygen species; MAPK, mitogen-activated protein kinase; JNK, Jun amino terminal kinase; ERK, extracellular signal-regulated kinase; NAC, *N*-acetylcysteine; TopII, opoisomerase II; PGC-1α, PPARG coactivator 1α; GPX1, glutathione peroxidase 1; HDAC, histone deacetylase; GATA4, GATA binding protein 4.

**Figure 2:**
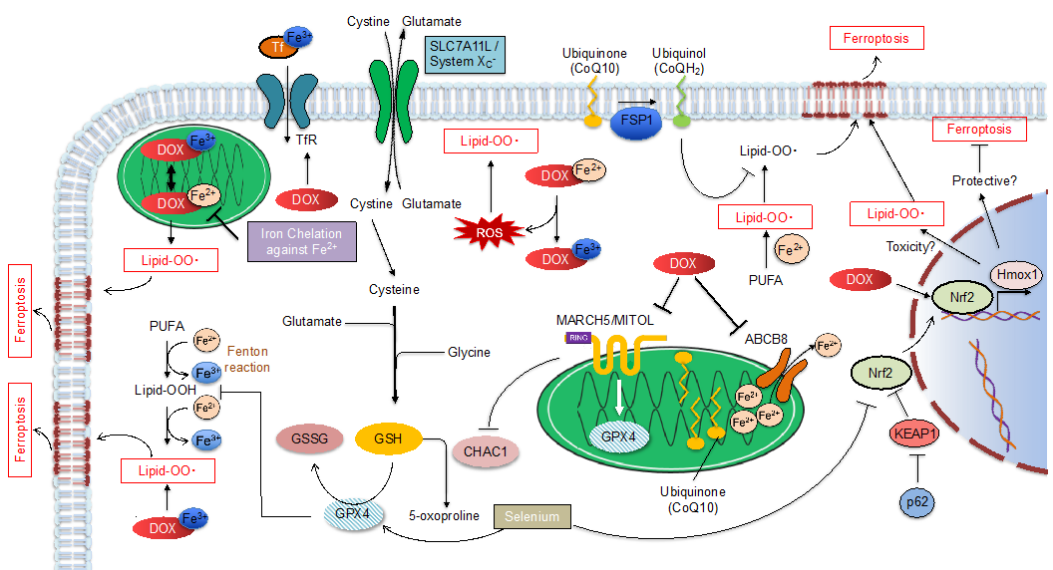
Schematic representation of Doxorubicin (DOX)-induced ferroptosis pathway. DOX, doxorubicin; Tf, transferrin; GSH, glutathione; GSSG, oxidized glutathione; GPX4, glutathione and glutathione peroxidase 4; FSP1, ferroptosis suppressor protein 1; CHAC1, ChaC Glutathione Specific Gamma-Glutamylcyclotransferase 1; Nrf2, Nuclear factor erythroid 2-related factor 2; KEAP1, Kelch-like ECH-associated protein 1; ABCB8, ATP-binding cassette transporter 8; CoQ10, coenzyme Q10.

Many basic studies have attempted to elucidate the molecular mechanisms of DOX cardiotoxicity, and approximately 400 studies have been published since 2000. Although apoptosis has been the focus of attention, as the final process leading to cardiomyocyte death, several recent studies have concluded that the newly proposed programmed death, ferroptosis, is involved in DOX-induced cardiomyocyte death. In this article, we review the reported mechanisms of DOX cardiotoxicity, broadly categorized into apoptosis (Figure 1) and ferroptosis (Figure 2), and classify them according to the molecular mechanism of each cell death.

### 1.1. Apoptosis

#### 1.1.1. DNA Damage

TopII has attracted attention as a classical cellular target of DOX [14]. There are two forms of the TopII enzyme: TopIIα and TopIIβ [15]. TopIIα is highly expressed in tumor cells, whereas TopIIβ is highly expressed in cardiomyocytes. TopIIβ-DOX-DNA ternary complex formation is important for DOX cardiotoxicity and induces DNA double-strand breaks in cardiomyocytes, resulting in cell death [16]. In the presence of TopIIβ, DOX decreases the transcription of genes, such as PPARG coactivator 1α (PGC1-α), involved in mitochondrial biogenesis and function [17]. Cancer patients with high expression levels of TopIIβ in cardiomyocytes are more sensitive to DOX cardiotoxicity, while mice lacking TopIIβ are resistant to DOX cardiotoxicity [17]. One reason for the increased cardiotoxicity risk with the combination of anthracyclines and trastuzumab is that trastuzumab changes the expression of the TopIIβ gene and protein in cardiomyocytes, leading to apoptosis [18]. Selective inhibitors of TopIIβ are a promising strategy to reduce toxicity to cardiomyocytes without compromising the potent anticancer effects of DOX. Dexrazoxane functions as a catalytic inhibitor of TopIIβ and reduces anthracycline-induced cardiotoxicity by suppressing DOX-induced DNA double-strand breaks [19]. Although concerns about second malignant neoplasms have been reported [20], dexrazoxane has been approved and clinically used in more than 30 countries to treat extravascular leakage of anthracycline antineoplastic agents.

#### 1.1.2. Oxidative Stress

Oxidative stress is one of the major mechanisms of DOX cardiotoxicity [21,22], and attenuating reactive oxygen species (ROS) by *N*-acetylcysteine and resveratrol is reportedly an efficient strategy for alleviating DOX-induced cardiotoxicity [23,24,25]. In the heart, after DOX administration, the major enzymes of the glycolytic system, triosephosphate isomerase, β-enolase, and ubiquinone oxidoreductase, which function as a transporter of electrons to the mitochondrial respiratory chain, were oxidized, and their activities decreased, suggesting that the bioenergetic pathway is an important target of DOX-induced oxidative stress [26]. There are many reports on the importance of the mitochondria as a source and target of oxidative stress [27,28,29]. The hearts of glutathione peroxidase (GPX) 1 knockout mice, scavenging H_2_O_2_ in the mitochondria and cytoplasm, showed significantly more severe DOX-induced cardiac dysfunction, mitochondrial injury, protein nitration, and apoptosis than the hearts of wild-type mice [30]. Although it is possible to enhance antioxidant stress mechanisms in cardiomyocytes by preconditioning with non-lethal oxidative stress stimuli, clinical application is difficult because similar cellular responses are expected to occur in cancer cells.

Research is also underway to establish iPS cells from patients and to differentiate them into cardiomyocytes to elucidate individual differences in sensitivity to DOX and the mechanisms involved. Human-induced pluripotent stem cell-derived cardiomyocytes (hiPSC-CMs) from breast cancer patients who experienced DOX-induced cardiotoxicity were consistently more sensitive to DOX toxicity, with decreased cell survival, impaired mitochondrial and metabolic function, impaired calcium handling, decreased antioxidant pathway activity, and increased production of ROS, compared to patients who did not experience DOX-induced cardiotoxicity [23]. These results suggest that individual differences in antioxidant mechanisms may define susceptibility to DOX. In fact, most recently, Chi Keung Lam et al. highlighted the importance of the use of patient-specific hiPSC-CMs in identifying populations who are at risk of drug-induced cardiotoxicity [31]. This strategy has potential applications in future precision medicine practice, especially in regard to the overlap cases between type I and type II drugs, such as sequential treatment with DOX and Her2 inhibitors, because the incidence of cardiotoxicity in this setting varies according to patient-related factors.

#### 1.1.3. Intracellular Signaling 

Among the intracellular signaling molecules, mitogen-activated protein kinase (MAPK) is one of the most studied target molecules for triggering DOX-induced cardiotoxicity. The MAPK cascade forms a signaling network that regulates cellular processes, including cell proliferation, growth, differentiation, transformation, and apoptosis. There are four major branching pathways of the MAPK pathway [32], and the p38 cascade is the main contributor to DOX-induced cardiotoxicity. Activated p38 induces apoptosis by acting on BAX, Bcl-2, and p53 [32,33,34,35,36]. A study examining the role of individual p38 isoforms, particularly the alternative isoforms p38γ and p38δ, in DOX-induced cardiotoxicity reported that p38δ plays an important role in promoting DOX-induced cardiotoxicity in females by inhibiting autophagy [37]. Conversely, many reports suggest that PI3k/Akt signaling, one of the most important kinases in the regulation of cell survival, has a significant effect on the inhibition of DOX-induced cardiotoxicity [38,39,40,41]. Indeed, the upregulation of the PI3k/Akt expression can inhibit apoptosis and improve DOX-induced cardiotoxicity [41,42,43]. Specifically, the hormone irisin, the membrane protein fibronectin type III domain-containing 5, and its cleavage substance, activate AKT/mTOR signaling and attenuate DOX cardiotoxicity-induced apoptosis [43]. In addition, some reports have suggested that JNK and ERK mediate DOX-induced cardiotoxicity [44,45]. Mechanistically, DOX treatment for rat myoblastic H9c2 cells shows increased phosphorylation levels of JNK and ERK, leading to the upregulation of NFkB, which further enhances cardiomyocyte apoptosis. Future studies to clarify the differences in the intracellular signaling mechanisms activated after treatment with DOX between cardiomyocytes and cancer cells will be useful for identifying target molecules that can mitigate cardiomyocyte toxicity while maintaining the anticancer effects of DOX.

#### 1.1.4. Transcription Factors 

In response to DNA damage, p53 induces cell cycle arrest and promotes DNA repair and apoptosis [46]. In DOX-induced cardiotoxicity, p53, activated by DNA double-strand breaks, triggers apoptosis. Dox-treated H9c2 cells showed increased phosphorylation of Ser15 in p53, the release of cytochrome c, and increased apoptosis [47]. In mouse hearts, DOX treatment promoted the acetylation of p53 protein and binding of p53 to the BAX promoter, upregulated BAX expression, and increased cytochrome c release from the mitochondria [48]. Using hiPSC-CMs, p53 was identified by RNA-seq as an upstream regulator of DOX-induced transcriptome changes [49]. Clustering and pathway analyses revealed that an increased expression of the death receptor and the exogenous apoptotic pathway were significantly associated with DOX-induced cardiotoxicity [49]. Suppression of the p53-mediated PGC1-α and APLNR (apelin receptor) signaling pathways limits energy production through fatty acid oxidation in the myocardium of DOX-treated mice [50]. DOX also inhibits histone deacetylases (HDACs) activity in a ROS-independent manner and promotes p53 acetylation [51]. Studies in p53-deficient mice have shown that the inhibition of p53 is cardioprotective during DOX treatment, but paradoxically leads to increased cardiotoxicity after the cessation of DOX treatment [52,53,54]. Other studies have shown that selective deletion of p53 in cardiomyocytes is insufficient to prevent the DOX-induced myocardial generation of reactive oxygen and nitrogen species, apoptosis, interstitial fibrosis, and perivascular fibrosis [55]. Thus, it is necessary to consider the possibility that the role of p53 in DOX-induced cardiotoxicity may differ between the acute and chronic phases. It has also been reported that DOX cardiotoxicity is associated with the reduced function of GATA binding protein 4 (GATA4), a transcription factor that regulates fetal cardiac development and stress response in adult cardiomyocytes [56]. By decreasing GATA4 levels, DOX downregulates Bcl2 expression and induces autophagy-related genes, leading to apoptosis and autophagy [57,58], and inhibits myofilament gene transcription, causing cardiac dysfunction due to extensive sarcomere structural abnormalities [59]. The establishment of animal models that separately investigate the mechanisms of acute and delayed cardiotoxicity after DOX treatment is warranted.

#### 1.1.5. Epigenetic Regulators 

Epigenetic modifications, including DNA methylation, histone modifications, and noncoding RNA expression, play an important role in regulating gene expression and contribute to DOX cardiotoxicity [60]. For instance, Ferreira et al. showed that pretreatment with DOX ameliorated H9c2 cells resistance against subsequent exposure to DOX [61]. They demonstrated that DOX pretreatment resulted in upregulation of mitochondrial DNA transcripts accompanied by a decrease in DNA methyltransferase 1 and global methylation levels. These results suggest that DOX pretreatment induces a beneficial and possibly epigenetic-based mitochondrial adaptation. Besides DNA methylation, histone modifications are also involved in DOX-induced cardiotoxicity. The expressions of several HDACs, such as HDAC6 and HDAC2, are affected by DOX treatment in cardiomyocytes [62,63,64]. For example, HDAC6 is upregulated in DOX-treated primary rat cardiomyocytes, resulting in deacetylation of α-tubulin [62]. On the other hand, HDAC2 is downregulated by DOX treatment, as well as decreased histone 3 acetylation [64]. Moreover, as mentioned above, another report showed that DOX cardiotoxicity leads to the suppression of the HDACs activity, which results in upregulation of p53 [51]. These data suggest that regulating these HDACs may also have a therapeutic potential for DOX cardiotoxicity. Furthermore, recent studies have demonstrated that noncoding RNAs, including long noncoding RNAs and microRNAs (miRNAs), are also involved in DOX cardiotoxicity [65,66,67,68]. Several miRNAs, such as miR-15 [65], miR-23a [68], and miR-34a [66,67], have been reportedly affected either in vitro or in vivo models of DOX-induced cardiomyopathy. In fact, overexpression of particular miRNAs, miR-232/132, showed substantial efficacy for DOX cardiotoxicity prevention in a mouse model [69]. Because epigenetic modification of the gene expression is a key early event in the transcriptional reprogramming of cardiac structural remodeling and metabolic flux in failing hearts [70], elucidating therapies to control this early step may be an important strategy in the future.

#### 1.1.6. Autophagy 

DOX is also known to exert toxic effects by affecting macroautophagy, chaperone-mediated autophagy, and the lysosome itself, the site where molecules transported by autophagy are degraded, thereby impairing proteolysis [71]. While some reports suggest that activating autophagy can inhibit DOX toxicity [72], others suggest that inhibiting dysregulated (or excessive) autophagy (or mitophagy) is necessary to reduce DOX toxicity [73,74]. Activating the Toll-Like Receptor 9/PI3Kγ pathway by DOX-damaged mitochondrial DNA in autolysosomes inhibits autophagy, thereby causing cardiac dysfunction [75]. Ghrelin inhibits DOX-induced autophagy and suppresses cardiomyocyte apoptosis by inducing mTOR via AMPK and p38-MAPK. [76] Resveratrol exerts its cardioprotective effect by inhibiting DOX-induced autophagy via S6K inhibition [77]. Bnip3, an autophagy-related molecule, interacts with LC3 on the outer mitochondrial membrane to sequester and degrade the mitochondria into autophagosomes [78]. In DOX-treated cardiomyocytes, Bnip3 expression was upregulated, inducing the opening of the mitochondrial permeability pore (mPTP) and loss of ∆Ψm, excessive mitochondrial fragmentation, mitophagy, and necrosis. Knockdown of Bnip3 prevents mitochondrial fission and DOX-induced cell death, suggesting that Bnip3 is involved in the cardiotoxicity of DOX [79,80]. Bnip3 is known to induce various forms of cell death depending on the cell situation, and there is a report that Bnip3 contributes to DOX-induced cardiomyocyte pyroptosis [79].

#### 1.1.7. Metabolic and Inflammation

SIRT1 protects cells from DNA damage and p53-dependent apoptosis [81] and also inhibits apoptosis by suppressing ROS production, p38 MAPK phosphorylation, and caspase-3 activation [82]. Many reports suggest that SIRT1 activation has a protective effect against DOX cardiotoxicity. Resveratrol activates SIRT1 and improves DOX cardiotoxicity [82,83,84]. AMPK also attenuates cardiotoxicity by activating SIRT1 [85,86,87]. In fact, calorie restriction and resveratrol exert protective effects against DOX cardiotoxicity via the activation of AMPK and SIRT1 and the induction of autophagy [88]. In addition, many reports show the inhibition of cardiotoxicity by activating SIRT1 through C1q/tumor necrosis factor-related protein-3 [89] and erythropoietin [90]. The p38- and p53-mediated pathways lead to apoptosis, but the pathway mediated by NF-κB is also important [91]. NF-κB activity inside and outside the mitochondria may affect mitochondrial dynamics, apoptosis, respiratory regulation, and gene expression [91]. Regarding DOX cardiotoxicity, several studies have shown that DOX activates NF-κB and causes apoptosis in cardiomyocytes, leading to cardiotoxicity [92,93,94]. As a therapeutic means of suppressing NF-κB and improving DOX cardiotoxicity, DHA [95], N-acetylcysteine [96], and berberine [97] alkaloids extracted from various herbs have been proposed. In contrast, it has been reported that DOX suppresses NF-κB signaling. Although DOX-induced upregulation of Bnip3 disrupts the homeostasis of mitochondrial dynamics and induces cell death, it has been reported that NF-κB signaling, which transcriptionally suppresses Bnip3 activation, is dramatically reduced after DOX treatment [98]. This report demonstrated that Bnip3 forms a protein complex with cyclophilin D and is involved in mPTP opening, leading to cell death.

### 1.2. Ferroptosis

Several studies have recently demonstrated that DOX-induced cardiotoxicity is caused by ferroptosis, a novel form of cell death [99,100,101,102,103,104,105]. Ferroptosis is a type of programmed cell death dependent on iron, and is characterized by the accumulation of lipid peroxides. The concept of ferroptosis was first reported by Dixon et al. in 2012 [106]. Since then, this programmed cell death has been implicated in various diseases [107,108,109]. In 2019, Fang et al. identified ferroptosis as the primary cause of cell death in DOX-induced cardiomyopathy [99]. They showed that only the ferroptosis inhibitor Ferrostatin-1 (Fer-1) significantly reduced DOX-induced mortality; in contrast, in mice treated with apoptosis, necroptosis, or autophagy inhibitors, survival was not significantly improved. DOX increases the nuclear accumulation of nuclear factor erythroid 2 -related factor 2 (Nrf2) and the expression of Hmox1 in cardiomyocytes, leading to the degradation of heme, resulting in the release of free iron and the formation of oxidized lipids in the mitochondrial membrane. Based on this report, the involvement of ferroptosis in the pathogenesis of DOX cardiomyopathy has attracted attention [100,101], and inhibiting this type of cell death with ferroptosis inhibitors, such as Fer-1, is expected to be a new therapeutic strategy [99,100,101]. Alpha-tocopherol, a well-known antioxidant that inhibits lipid peroxidation [110] also reduces DOX cardiotoxicity [111,112,113].

#### 1.2.1. Apoptosis or Ferroptosis?

A new form of cell death called ferroptosis was identified after looking back on previous reports, and it was concluded that apoptosis is the central mechanism of DOX-induced cardiotoxicity. However, even among studies concluding that the final form of cell death is apoptosis, many reports are suggesting the involvement of iron in the process of cell death. For example, one trial of patients with advanced breast cancer revealed that dexrazoxane, which is the only drug that is FDA approved to prevent DOX cardiotoxicity, has a significant cardioprotective effect [114]. Mechanistically, dexrazoxane is a chelator of intracellular iron, which blocks iron-assisted oxidative radical production, suggesting the importance of iron overload in the heart as a mechanism caused by DOX [115,116]. In bovine aortic endothelial cells, iron uptake into the cells is markedly increased during DOX-induced cell death, and iron regulatory protein-1, a central cytosolic regulator of cellular iron metabolism, is activated [117]. This DOX-induced iron uptake occurs through a transferrin receptor-dependent mechanism, and the administration of anti-transferrin receptor antibodies dramatically suppress DOX-induced iron uptake, intracellular oxidant production, and cell death. In clinical practice, serum transferrin levels in patients receiving DOX chemotherapy correlate with left ventricular dysfunction severity [118]. Treatment of the HL-1 cell line with DOX caused a time-dependent increase in cytoplasmic and mitochondrial free iron pools, resulting in a loss of mitochondrial membrane potential [102]. Ferritin sequesters iron, consequently protecting cells against iron-mediated free radical damage. Mice lacking mitochondrial ferritin localized in the mitochondrial matrix exhibited enhanced DOX-induced cardiotoxicity [119]. In addition, another study demonstrated that DOX becomes concentrated in the mitochondria and increases both mitochondrial iron and cellular ROS levels [120]. The ATP-binding cassette (ABC) transporter ABCB8, localized in the mitochondrial inner membrane, is responsible for mitochondrial iron export and plays an essential role in maintaining mitochondrial iron homeostasis and maturation cytosolic Fe/S proteins [121]. An overexpression of ABCB8 in the heart reduces mitochondrial iron and intracellular ROS levels and suppresses DOX-induced cardiomyopathy [120].

Understanding the difference between these two programmed cell death pathways is important to determine whether the underlying mechanism of DOX-induced cardiotoxicity is apoptosis or ferroptosis (Table 2). In fact, the morphological changes and cell death cascade induced by apoptosis are completely different from those of ferroptosis [106]. In ferroptosis, chromatin aggregation is not observed as in apoptosis. Moreover, caspase activation was not observed during ferroptosis [122,123]. In DOX, cardiotoxicity, apoptosis, and cell death due to ferroptosis may occur simultaneously [100,101,124]. In our study, when primary cultured rat myocardial cells were treated with 5 μM DOX for 24 h, cell death caused by low-dose DOX treatment could be rescued by either the apoptosis inhibitors ZVAD or Fer-1. However, the inhibitory effect of Fer-1 on cell death was stronger than that of ZVAD [100]. Ferroptosis inhibitors do not alter caspase activation or the amount of cytochrome c in the cytosol, representing mitochondria-dependent apoptosis. In contrast, the apoptosis inhibitor ZVAD does not reduce the number of lipid peroxides that cause ferroptosis [101,124]. This result might indicate that both apoptosis and ferroptosis present DOX toxicity as independent cell death that does not affect each other. As Table 2 demonstrates, there are several apoptosis or ferroptosis inhibitors other than ZVAD and Fer-1. For instance, Emricasan, Q-VD-Oph, IDN-6556, and DEVD-CHO have been used as apoptosis inhibitors in several studies [103,125]. While Emricasan failed to show protective effects for DOX-induced cardiotoxicity [99], other apoptosis inhibitors, including Q-VD-Oph and DEVD-CHO, have not been investigated well in DOX cardiotoxicity. On the other hand, as ferroptosis inhibitors, vitamin E (α-tocopherol) and liproxstatin-1 are well-known radical trapping agents. In fact, vitamin E had been reported to play an effective antioxidant role to prevent apoptosis before ferroptosis was first reported. Indeed, as mentioned above, previous reports showed that vitamin E decreased doxorubicin-induced cardiotoxicity [113]. Although there is a scarcity of data that directly show the liproxstatin-1 efficacy for DOX cardiotoxicity, these radical trapping agents, including Fer-1, may be potential targets for DOX cardiotoxicity prevention.

In addition, DOX activates necroptosis, which, together with necrosis, causes more cell death than apoptosis [102,128]. DOX upregulates RIPK3, which binds to and phosphorylates calmodulin kinase II, which in turn regulates the opening of mPTP, leading to necroptosis and apoptosis [102]. Thus, various types of cell death are involved in DOX-induced cardiotoxicity, and the mechanisms of each type of cell death at least partially overlap.

#### 1.2.2. Ferroptosis Regulatory Pathway

##### Glutathione Peroxidase 4 (GPX4)

GPX4 is a selenium-containing enzyme that uses reduced glutathione (GSH) as a co-factor to reduce lipid peroxide accumulation [129]. GPX4 is considered to be the primary enzyme that prevents ferroptosis. The ferroptosis activator RSL3 (1S,3R) induces ferroptosis by inhibiting GPX, which contains the essential microelement selenium, particularly GPX4 [130]. Tadokoro et al. demonstrated that DOX downregulates GPX4 expression and induces excessive lipid peroxidation via the mitochondrial DOX-Fe^2+^ complex, leading to mitochondria-dependent ferroptosis [101].

##### MITOL/MARCH5

As mentioned above, DOX downregulates the GPX4 expression and causes ferroptosis. However, the reason DOX decreases the expression of GPX4 is unclear. We found that the depletion of GSH in cardiomyocytes caused a decrease in the expression of GPX4 in the mitochondria [100]. MITOL/MARCH5 is an E3 ubiquitin ligase that plays an important role in regulating mitochondrial quality and function. In primary cultured neonatal rat cardiomyocytes, downregulation of MITOL expression by siRNA markedly reduced GPX4 localized in the mitochondria, promoting the accumulation of lipid peroxides in the mitochondria and induced cell death by ferroptosis. In MITOL-knockdown cells, the glutathione-degrading enzyme ChaC Glutathione Specific Gamma-Glutamylcyclotransferase 1 (CHAC1) expression was upregulated, resulting in a decrease in the glutathione/oxidized glutathione (GSH/GSSG) ratio. Improving the GSH/GSSG ratio through the administration of *N*-acetylcysteine or knockdown of CHAC1 suppressed ferroptosis by recovering the expression of GPX4 in MITOL knockdown NRVMs. In DOX-treated cultured cardiomyocytes, both MITOL and GPX4 decreased, but the forced expression of MITOL maintained the GPX4 content and prevented DOX-induced ferroptosis. Sensitivity to DOX cardiotoxicity increased in mice lacking MITOL. These results suggest that MITOL plays a role in protecting cardiomyocytes from DOX-induced toxicity by regulating mitochondrial glutathione levels and GPX4 expression. As ferroptosis caused by MITOL knockdown is a cardiomyocyte-specific phenomenon, the authors believe that it may explain at least part of the mechanism through which cardiomyocytes are more dysfunctional after DOX treatment than other cells. If it is possible for us to examine the expression of MITOL in the heart, we may be able to conduct risk stratification of cardiotoxicity caused by DOX. Moreover, compounds that upregulate MITOL may be a promising strategy to mitigate DOX-induced cardiotoxicity. Other studies have shown that the administration of GSH and glutamate, necessary for GSH synthesis, can reduce the cardiotoxicity of DOX [131,132,133]. GSH, a co-factor of GPX4, is a tripeptide of cysteine, glutamate, and glycine. Cysteine is often depleted in the cells. Cystine/glutamate antiporter solute carrier family 7 member 11 (SLC7A11, also known as System X_c−_) promotes cystine uptake and glutathione biosynthesis. Erastin and sulfasalazine inhibit GSH synthesis by inhibiting SLC7A11, leading to ferroptosis [134].

##### Nrf2

Nrf2 and its repressor Kelch-like ECH-associated protein 1 (KEAP1) are key metabolism, oxidative stress, and inflammation regulators. In recent years, several reports have advocated the regulation of DOX cardiotoxicity by Nrf2 [135,136,137]. Bardoxolone methyl, an activator of the Nrf2-KEAP1 pathway and a potential treatment for chronic kidney disease, reduces DOX-induced cardiotoxicity in 3D cardiac spheroids, in which human cardiomyocytes are grown in combination with human cardiac fibroblasts and endothelial cells. Exposure of hepatocellular carcinoma cells (HCC) to ferroptosis-inducing compounds, such as elastin and sorafenib, promotes the nuclear accumulation of Nrf2 through the inactivation of KEAP1 by p62 expression. Knockdown of p62, quinone oxidoreductase-1, Hmox-1, and ferritin heavy chain-1 promotes ferroptosis in HCC cells in response to elastin and sorafenib [107]. The ubiquitin E3 ligase TRIM21 interacts with p62 and ubiquitinates it, negatively regulating the p62-KEAP1-Nrf2 antioxidant pathway [138]. Cardiac tissue lacking TRIM21 is protected from DOX-induced ferroptosis by homeostatic activation of Nrf2. As mentioned above, selenium inhibits ferroptosis by promoting the expression of the antioxidant GPX4; however, selenium also exerts a protective effect against DOX-induced cardiotoxicity by inhibiting the Nrf2-NLRP3 pathway [139]. It has been reported that Hemin stimulates Nrf-2/Hmox-1 and inhibits the TLR-5/NF-κB/TNF-α pathway, thereby exerting antioxidant and anti-inflammatory effects and reducing DOX-induced cardiotoxicity in a dose-dependent manner [140]. In contrast, there are reports that Nrf2 activation is involved in ferroptosis induction. Hassannia et al. reported that elevated Nrf2 activates Hmox1, increasing the amount of unstable Fe(II) ions, resulting in lipid peroxides production, ultimately inducing ferroptosis [141]. Fang et al. also concluded that DOX causes an increase in Nrf2, leading to cardiotoxicity via a similar mechanism [99].

##### Ferroptosis Suppressor Protein 1 (FSP1)

Another known defense mechanism against ferroptosis is FSP1, a GPX4- and GSH-independent ubiquinone reductase [142,143]. FSP1 was described as a pro-apoptotic gene apoptosis-inducing factor mitochondria-associated 2 (AIFM2) before it was renamed. The reduced ubiquinol scavenges peroxyl radicals (ROO•) causing lipid peroxidation. FSP1 uses NAD(P)H to catalyze the regeneration of ubiquinone (coenzyme Q10 (CoQ10)). To date, no evidence has been published regarding the direct relationship between FSP1 and DOX cardiotoxicity, but the fact that the administration of CoQ10 minimized DOX-induced toxicity, such as ECG changes, oxidative stress, lipid peroxidation, and changes in cardiac tissue, may suggest an association between DOX-induced cardiotoxicity and FSP1 [144,145].

## 2. Mitochondria Providing a Place for DOX-Induced Cardiotoxicity

Whether the mechanism of DOX cardiotoxicity is apoptosis or ferroptosis, the mitochondria are important places where toxicity is exerted [146,147,148,149,150,151,152,153,154,155]. It has been theorized that mitochondria-mediated apoptosis (endogenous pathway) is one of the mechanisms of DOX cardiotoxicity [147,150,156,157,158,159,160,161,162,163]. Mitochondria also play a central role in ferroptosis induction [99,100,101]. DOX disrupts protein complexes between major respiratory chain proteins, such as uncoupling protein 3 and cytochrome c oxidase, and induces abnormal mitochondrial respiration and various types of cell death through a Bnip3-dependent mechanism [80,98,162]. Mitochondria retain their homeostasis through repeated fission and fusion. DOX fragments mitochondria by inducing the activation of dynamin protein 1 (Drp1), which is important for mitochondrial fission [157,163,164]. Conversely, the induction of the mitochondrial fusion factor Mitofusin2 by DOX acts protectively against DOX-induced toxicity [164]. In addition, the involvement of mitochondrial fission protein 1 (Mtfp1), a new molecule regulating mitochondrial dynamics, has also been proposed [165]. Mechanistically, DOX upregulates the Mtfp1 expression, which induces mitochondrial fission and apoptosis. Moreover, in DOX-treated cardiomyocytes, the decreased expression of a long non-coding RNA called CMDL-1, which is involved in post-translational modification (phosphorylation) of Drp1, has also been reported to be associated with Drp1-induced promotion of mitochondrial fission and cell death [166]. Collectively, there is no shortage of evidence suggesting the importance of mitochondria in DOX cardiotoxicity.

## 3. Conclusions

This article summarizes the mechanisms involved in DOX cardiotoxicity, mainly related to two forms of cell death, apoptosis and ferroptosis, by focusing on the basic research in this century. Numerous previous studies suggest that various types of cell death are involved in DOX cardiotoxicity. The underlying mechanisms that induce multiple types of cell death and their common regulators may serve as therapeutic targets to alleviate DOX-induced cardiotoxicity. We await the results of basic research that will lead to a therapeutic strategy to mitigate cardiotoxicity, while maintaining the high anti-tumor efficacy of DOX or inhibiting the progression of delayed cardiac dysfunction after cancer eradication.

## Figures and Tables

**Table 1:** Characteristics of type I and II chemotherapy-induced cardiotoxicity.

	Type I (Myocardial Damage)	Type II (Myocardial Dysfunction)
Representative agents	Anthracyclines (DOX)	Trastuzumab
		Bevacizumab
		Sunitinib
		Sorafenib
Dose-dependence	Yes	No
Mechanism	Free radical formation	Inhibition of Erb signaling
	DNA damage	
	Oxidative damage	
Clinical manifestation	Underlying damage appears to be permanent and irreversible	High likelihood of recovery (to or near baseline cardiac status) in 2–4 months (reversible)
Biopsy presentation	Myofibril disarray	Minimal changes have been reported
	Vacuole formation	
Effect of rechallenge	High probability of recurrent dysfunction that is progressive, leading to intractable heart failure and death	Increasing evidence for the relative safety of rechallenge; additional data needed

Modified from reference [2,9].

**Table 2:** Differences between features of apoptosis and ferroptosis.

	Apoptosis (Intrinsic Apoptosis)	Ferroptosis
Biochemical characteristics	Involvement of Bcl-2 family proteins Release of cytochrome c from mitochondria Activation of caspases	Peroxidation of cell membrane phospholipids catalyzed by iron ions (Fe^2+^) Accumulation of lipid hydroxyl radicals
Key molecules	Caspase-3, Bcl-2, BAX, p53, NF-κB	GPX4, GSH, Xc−, CHAC1, CoQ10, Nrf2
Inhibitors	ZVAD-FMK, Emricasan, Q-VD-Oph, IDN-6556, DEVD-CHO	Fer-1, Vitamin E, Liproxstatin-1, CoQ10
Morphological features	Chromatin condensation and fragmentation DNA laddering Cell shrinkage and bleb formation at the plasma membrane Exposure of phosphatidylserine to the outer membrane	Decrease in mitochondrial cristae Mitochondrial aggregation

Modified from reference [125,126,127].

## Data Availability

Not applicable.
